# Investigating the Health Impacts of Climate Change among People with Pre-Existing Mental Health Problems: A Scoping Review

**DOI:** 10.3390/ijerph20085563

**Published:** 2023-04-18

**Authors:** Lisa Woodland, Priyanjali Ratwatte, Revati Phalkey, Emma L. Gillingham

**Affiliations:** 1 Department of Psychological Medicine, King’s College London, London SE5 9RJ, UK; 2 NIHR Health Protection Research Unit in Emergency Preparedness and Response, King’s College London, London SE5 9RJ, UK; 3 Climate Change and Health Unit, UK Health Security Agency, Chilton OX11 0RQ, UK; 4 Behavioural Science and Insights Unit, UK Health Security Agency, Porton Down, Salisbury SP4 0JG, UK

**Keywords:** extreme heat, flood, wildfire, hurricane, drought, depression, anxiety, post-traumatic stress, dementia, schizophrenia

## Abstract

Climate change is the greatest threat to global public health, although the impacts on mental health are relatively understudied. Furthermore, there is a lack of consensus about the effects of climate change on individuals with pre-existing mental health problems. This review aimed to identify the health impacts of climate change on people with pre-existing mental health problems. The search was conducted across three databases; studies were included if they involved participants who had mental health problem(s) before a climate-driven event and reported on health outcomes post-event. A total of thirty-one studies met the full inclusion criteria. The study characteristics included 6 climate-driven events: heat events, floods, wildfires, wildfire and flood, hurricanes, and droughts, and 16 categories of pre-existing mental health problems, with depression, and non-specified mental health problems being the most common. The majority of the studies (90%, n = 28) suggest an association between the presence of pre-existing mental health problems and the likelihood of adverse health impacts (e.g., increased mortality risk, new symptom presentation, and an exacerbation of symptoms). To mitigate the exacerbation of health inequalities, people with pre-existing mental health problems should be included in adaption guidance and/or plans that mitigate the health impacts of climate change, future policy, reports, and frameworks.

## 1. Introduction

Climate change is the greatest threat to global public health [1]. According to the 2022 Global Report of the Lancet Countdown about climate change and health, every continent experienced major destruction from climate-driven events during 2021 and 2022 [2]. In 2021, economic losses associated with climate-driven events totalled US$253 billion, with 84% occurring in high-Human-Development-Index (HDI) countries [2]. Climate change exacerbates health-related vulnerabilities; the determinants of vulnerability relate to exposure to climate change stressors, the degree to which people or communities are predisposed to be adversely affected by climate change, and a lack of advanced capacity to cope and adapt to impacts [3,4,5]. The effects of climate change can range from morbidity and mortality relating to climate-driven events; malnutrition associated with declining food security; increased incidence of infectious diseases, particularly vector-borne and water-borne illnesses; increased respiratory and cardiovascular disease; mental health impacts; and impacts on healthcare system resilience [5,6,7,8]. These impacts are already observable, demonstrated by the increased incidence and severity of climate-driven events, such as extreme heat, flooding, wildfires, and drought [9].

To be able to cope with normal life stressors and to function properly, individuals need to have good mental health [10]; mental health refers to an individual’s emotional, psychological, and social well-being. Whilst the impacts of climate change on physical health are well-documented, its effects on mental health have been relatively understudied, although understanding and addressing mental health impacts have been identified as a critical priority to protect and improve human health [2]. For example, during the first year of the COVID-19 pandemic, there was a 25% increase in anxiety and depressive disorders globally, and the treatment gap for mental health conditions widened [11]. Similarly, more than 33 million people were affected by the 2022 floods in Pakistan, with an estimated 50% of children and their caregivers reporting signs of distress; there were inadequate mental health services to support their mental health needs [12,13]. Finally, during 2022, there were over 7500 wildfires reported in California (USA), and people directly affected by the fires were at higher risk of depression and post-traumatic stress disorder (PTSD) [14]. A systematic review of the health impacts of climate change identified several pathways in which climate change may impact mental health [8]. High temperatures were associated with adverse mental health outcomes, increased hospital admissions for mental health-related reasons, increased incidence of suicide, exacerbation of pre-existing mental health conditions, and difficulty sleeping and fatigue [8]. Furthermore, flooding and drought were associated with psychological distress, PTSD, anxiety, depression, and substance and alcohol misuse [8]. Only 14% of studies included in the systematic review reported on mental health outcomes, which does not necessarily suggest that the mental health impacts of climate-driven events are lower than the physical health impacts. Instead, this finding highlights that research about climate-driven events and mental health is limited. Vulnerable populations, such as people from low-income households, people with chronic illness and disabilities, older adults, ethnic minorities, indigenous people, and people experiencing housing instability, amongst others, are disproportionately affected by the health impacts of climate change, including mental health impacts [2,15], which exacerbate pre-existing mental health inequalities.

A particularly understudied vulnerable group are people with pre-existing mental health problems. Pre-existing mental health problems have been identified as a vulnerability factor by the WHO [11] and have been highlighted by several other studies as vulnerable to the exacerbation of health problems [16,17,18]. People with pre-existing mental health problems struggle to cope during and after climate events because of disruptions to mental health services and a lack of resiliency and resources [19], which reduces the ability to cope with the event [20,21]. There is a lack of detailed consensus on the potential health impacts of climate-driven events on people with pre-existing mental health problems, including the exacerbation of pre-existing mental health problems.

Understanding the health impacts on those with pre-existing mental health problems is important for policy-makers, practitioners, and researchers so that the risks can be incorporated into research and policy to mitigate the health risks to these individuals who are already vulnerable. The incidence of mental health problems is increasing worldwide, being a major contributor to the global disease burden [22,23]. It has been estimated that one in four people will experience a mental health condition in their lifetime [24]; therefore, understanding how climate change impacts people with pre-existing mental health problems should be prioritised to strengthen adaptation strategies and achieve co-benefits for adaptation/mitigation and health protection.

This scoping review aimed to understand the health impacts of climate-driven events on individuals with pre-existing mental health problems and to identify gaps in the existing literature.

## 2. Materials and Methods

### 2.1. Search Strategy and Selection Criteria

The search was conducted using three databases, PsycInfo, Embase, and Medline. The search was started on the 20th and was completed on 25thApril 2022. The search strategy used a combination of climate-driven events (e.g., “heat”, “wildfire”, and “drought”) AND mental health terms and disorders (e.g., “well-being”, “mental health”, and “depression”). Truncation and wildcards were used on search terms, such as schiz, * to include schizophrenia and schizophrenic in the search. Different terms for the same climate-driven event and mental health problem were also used, such as “post traumatic stress”, “post-traumatic stress”, and “PTSD”. The search strategy can be found in Supplementary Materials Text S1. All records were imported into Endnote (version 20) [25], and duplications were removed. The citations of included papers were also reviewed.

The study used Arksey and O’Malley’s five-stage framework about how to conduct a scoping review [26], as well as the PRISMA extension for scoping reviews (PRISMA-ScR) [27], which is a checklist of 22 items that should be reported in scoping reviews. Both PRISMA-ScR and Arksey and O’Malley’s framework were used because they draw on similar features and cover items that are omitted from one of the report guides.

### 2.2. Inclusion Criteria

Studies were included that investigated whether there was a change in the mental health status of the participants with pre-existing mental health problems after exposure to a climate-driven event. To be included in the review, the study had to state that the participants had pre-existing mental health problems, for example, by conducting surveys before a climate-driven event or by asking participants about their mental health history retrospectively after an event. Both qualitative and quantitative studies written in English were included in the study, whilst studies that did not include original data, such as reviews, letters, commentaries, and book chapters, were excluded. Only studies published since the year 2000 were included, as there were a limited number of studies published, and some of the authors repeated the studies after 2000.

### 2.3. Data Extraction and Synthesis

Data were extracted from the included studies by the primary author, and the extracted data can be found in Table A1, which includes relevant data about the included studies (climate-driven event, country, study design, sample characteristics, study period, pre-existing mental health problem, and reported impacts on health). The reported health outcome data included the effect measure(s) that directly relate to the study measures, such as the risk ratio (RR) and odds ratio (OR) in quantitative studies, and description of the health impacts in qualitative studies. A second author also reviewed a sample of the included and excluded papers to ensure that a consistent method was used, and three authors reviewed all the data that were extracted from the included studies. 

The included studies were narratively synthesised due to the expected heterogeneity in the study designs and outcomes. The included studies were grouped by climate-driven event and the health impacts that relate to our study’s aims were reported for each study. The included studies were synthesised by pre-existing mental health problem so that observations could be made about the impacts of the climate-driven event on people with the specified pre-existing mental health problem.

## 3. Results

### 3.1. Study Selection

**Figure 1:**
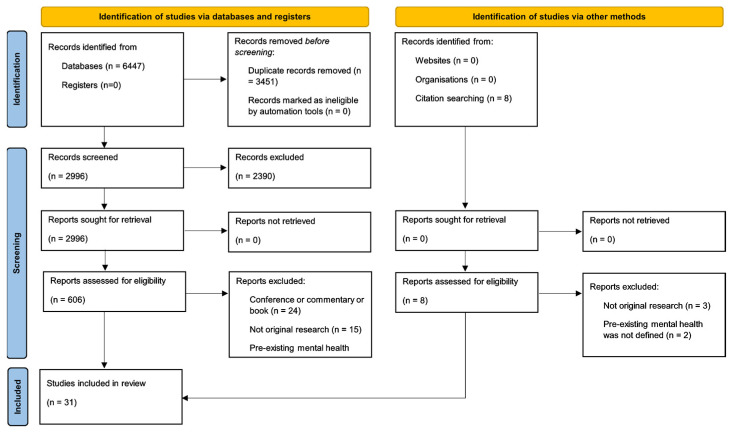
PRISMA 2020 flow diagram of studies included in the review to investigate the mental health impacts of climate-driven events among people with pre-existing mental health problems [28].

The initial search yielded 6447 research studies, and 3451 were duplicates. The titles of 2996 studies were screened and 606 abstracts and full texts were assessed against our inclusion criteria. A further eight studies that had been identified through citation searches were assessed against our inclusion criteria. In total, 31 studies were included in the review (Figure 1).

### 3.2. Study Characteristics

Of the 31 included studies, the majority reported on heat events (39%, eight papers [29,30,31,32,33,34,35,36]; four case studies [37,38,39,40]), followed by floods (26%, seven papers [41,42,43,44,45,46,47]; one thesis [48]), wildfires (23%, seven papers [49,50,51,52,53,54,55]), wildfire and flooding (3%, one paper [56]), hurricanes (6%, one paper [57]; one thesis [58]), and droughts (3%, one paper [59]). The studies were conducted in nine different countries/regions: the USA (35%, n = 11) [30,32,41,42,45,46,47,48,57,58,59]; Canada (26%, n = 8) [38,44,50,51,52,53,54,55]; Italy (10%, n = 3) [29,34,35]; Australia (6%, n = 2) [49,56]; Brazil (6%, n = 2) [36,37]; England (6%, n = 2) [33,43]; Hong Kong (3%, n = 1) [39]; South Korea (3%, n = 1) [31]; and Taiwan (3%, n = 1) [40] (rounding errors account for 2%). Twenty-six studies (84%) were quantitative [29,30,31,32,33,34,35,36,41,42,44,45,46,47,48,49,50,51,52,53,54,55,56,57,58,59] and five (16%) were qualitative (including case studies) [37,38,39,40,43]. Full results detailing the findings of each study are presented in Table A1.

As the terms used to describe mental health problems varied between papers, we combined terms of similar mental health problems. Herein, ‘depression’ includes studies that discussed depression symptoms, depression, and major depressive disorder; ‘anxiety’ includes studies that discussed anxiety and general anxiety disorder; ‘schizophrenia and comorbidities’ describes participants with either schizophrenia and bipolar or schizophrenia and diabetes mellitus; ‘dementia’ includes dementia and dementia and cognitive decline; and ‘substance misuse’ includes alcohol and substance (mis)use. If studies did not investigate a specific mental health problem, but, for example, asked if they had engaged with a “professional for a mental health concern” [49], the pre-existing mental health problem was defined as ‘non-specified.’ On occasions, studies investigated multiple specific mental health problems, but grouped the results together (e.g., people with psychosis, dementia, and alcohol and other substance misuse were grouped together as the study’s health outcome [33])—we defined these as ‘aggregated’ mental health problems. Table A1 reports the specific mental health problems included in the ‘aggregated’ category studies.

A total of 16 categories of pre-existing mental health problems were identified: depression (48%, n = 15) [29,32,34,35,41,42,43,44,45,48,50,51,53,54,55]; non-specified (23%, n = 7) [32,35,49,50,52,56,59]; anxiety (19%, n = 6) [44,50,51,53,54,55]; aggregated (19%, n = 6) [30,31,33,35,46,47]; dementia (10%, n = 3) [33,35,43]; psychosis (10%, n = 3) [29,33,34]; schizophrenia (6%, n = 2) [37,40]; schizophrenia and comorbidities (6%, n = 2) [38,39]; personality disorders (6%, n = 2) [35,43]; substance misuse (6%, n = 2) [33,35]; mania and bipolar (3%, n = 1) [35]; attention-deficit hyperactivity disorder (ADHD) (3%, n = 1) [58]; neurotic disorders (3%, n = 1) [35]; obsessive compulsive disorder (OCD) (3%, n = 1) [36]; chronic mixed anxiety and depression (3%, n = 1) [43]; and post-traumatic stress disorder (PTSD) (3%, n = 1) [57].

Some studies reported more than one health outcome after a climate-driven event (Table 1): 90% (n = 28) of studies demonstrated adverse mental health impacts and 42% (n = 13) of studies showed no health impacts, which included two studies (6%) that had inconclusive results (the same study reported adverse health impacts and no health impacts) on participants with pre-existing mental health problems.

### 3.3. Heat Events

Twelve studies (including four case studies indicated with *) investigated the impacts on mental health of heat events, which included heatwave events [30,31,32,37,38,40] *, exposure to temperatures exceeding a defined threshold [29,33,34,35], or exposures to high temperatures [36,39] *. Studies investigated heat events in eight countries/regions: Italy [29,34,35], Brazil [36,37] *, USA [30,32], Canada [38] *, England [33], Hong Kong [39] *, South Korea [31], and Taiwan [40] *. Studies (excluding case studies) measured the daily air temperature over a period ranging from 9 days [32] to 14 years [35]. Eleven pre-existing mental health problems were reported: depression [29,32,34,35]; mania and bipolar [35]; psychosis [29,33,34]; schizophrenia [37,40] *; schizophrenia and comorbidities [38,39] *; neurotic disorders [35]; disorders of personality and behaviour [35]; dementia [33,35]; OCD [36]; substance misuse [33,35]; non-specified [32,35]; and aggregated [30,31,33,35] (Table 1). Eight studies measured mental health before the onset of a heat event [29,33,34,35,37,38,39,40] * whilst four studies asked participants to retrospectively report previous mental health problems during the course of the study [30,31,32,36]. There was one study that investigated children and adults [31], one study that did not specify whether children and/or adults were participants [33], and the remaining ten studies investigated adults only.

Of the four studies that investigated the effect of heat events on people with depression, three studies found that individuals with depression were at a significantly increased risk of mortality compared with participants without depression during high air temperatures [32,34,35], whilst mixed results were reported in the fourth study [29]. First, during a heatwave in the USA (Chicago), participants with depression were at a four-times-higher risk of mortality compared with those without depression, based on the death certificates of participants where heat was the primary or secondary cause of death [32]. Second, an investigation into mortality risk factors in participants from four Italian cities found an increased mortality risk in participants with depression at 30 °C compared with 20 °C [34], whilst another study focusing on participants with depression in an Italian city (Bologna) found an increased mortality risk per 1 °C increase above 24 °C [35]. In contrast, an increased mortality risk in participants with depression was reported in only one of six different locations in three Italian cities (Milan, Rome, and Turin), with no increased risk detected in the other five locations: the only location where there was an increased mortality risk experienced the highest daily mean air temperature compared with the other five locations [29].

Of the three studies that investigated mortality risk for people with psychosis following exposure to high temperatures, the findings were mixed. First, participants in four Italian cities with psychosis were at a 1.70-times-higher risk of mortality when the air temperature was 30 °C compared with when it was 20 °C [34]. In addition, the study reported above that measured mortality risk at six different locations in Italy found that participants with psychosis were at 91%, 93%, and 157% increased risk of mortality compared with participants without psychosis in three locations, but there was no significant effect measured in the remaining three locations [29]. The final study reported no increased risk of mortality for participants with psychosis in England following exposure to temperatures above 18 °C [33].

Four case studies described four male participants with a history of schizophrenia [37,40] *, or schizophrenia and comorbidities (schizophrenia and bipolar [38] *; schizophrenia and diabetes mellitus [39] *), and their admission to hospital with heatstroke following exposure to high temperatures. On admission, all four participants were assessed using the Glasgow Coma Scale (GCS), a measure (out of 15) of a person’s level of coma or consciousness. The patient with the least severe heatstroke diagnosis scored 14 on the GCS and was described as drowsy, weak, and slurring on admission [40] *. The next-most-severe patient had a GCS of 9 and was diagnosed with heatstroke [37]. The most severe cases scored 3 on the GCS on admission: one patient was described as unresponsive [38] * and the other as comatose [39] *, and both patients were intubated. In all four cases, the authors suggested that the medication the patients were on may have induced or exacerbated the health-related illness.

Of the two studies that reported the impact of exposure to high temperatures on dementia patients, both studies found significantly increased mortality risk for participants with dementia. One study in England found that mortality risk increased by 1.03 for every 1 °C above 18 °C compared with the controls [33]. The second study found that mortality risk in dementia patients in Italy increased by 1.07 for every 1 °C above 24 °C compared with those without dementia [35].

The two studies investigating heat events on participants with substance misuse reported contrasting results. One study in England found that for every 1 °C increase above 18 °C, the risk of mortality increased by 1.08 for participants with alcohol misuse and by 1.20 for participants with substance misuse compared with controls [33]. In contrast, one Italian study found no difference in mortality risk for participants with alcohol and substance misuse compared with the controls [35].

Two pre-existing mental health problems were focused on in only one study reporting the impacts for participants with each health problem and heat. One study investigated the impact of weather on people with OCD in Brazil, finding that hot weather exacerbated their OCD symptoms [36]. The second study found no difference in mortality risk for participants with pre-existing neurotic disorders, personality and behaviour disorders, and mania and bipolar compared with participants without mental health problems [35].

Of the two studies that investigated non-specified mental health problems and mortality risk, reported contrasting results. One study found that, during a heatwave in the USA (Chicago), participants with non-specified mental health problems were at a 11.7-times-higher risk of heat-related death than controls [32]. In contrast, a study in Italy (Bologna) found no increased risk for participants with pre-existing non-specified mental health problems and mortality during temperatures above 24 °C [35].

Of the four studies that investigated exposure to heat and health impacts for participants with aggregated mental health problems, three studies reported health impacts and one reported no significant effects on health. First, the mortality risk for participants with pre-existing aggregated (psychosis, dementia, and substance misuse) mental health problems was 1.05 times higher for every 1 °C above 18 °C compared with participants without mental health problems [33]. A second study found the risk of mortality was 14 times higher for participants with pre-existing aggregated (schizophrenia, mentally handicapped, dementia, and alcohol abuse) mental health problems [30]. A third study found that participants with aggregated (schizophrenia, dementia, depression, Parkinson’s disease, panic disorders, bipolar disorder, substance misuse, mental retardation, and unknown mental health diagnosis) mental health problems were at a 7.69-times-higher risk of heatstroke compared with participants without mental health problems during periods of high air temperatures [31]. In contrast, one of the studies found no increased risk for participants with aggregated (schizophrenia and other functional psychosis) mental health problems compared with those without [35].

### 3.4. Floods

There were eight studies covering three countries (the USA [41,42,45,46,47,48], Canada [44], and England [43]) that reported on floods. Six pre-existing mental health problems were reported: depression [41,42,43,44,45,48]; anxiety [44]; chronic mixed anxiety and depression [43]; paranoid personality disorder [43]; dementia [43]; and aggregated [46,47] (Table 1). Five studies measured mental health before the floods [41,42,44,45,48], whilst three studies retrospectively measured pre-existing mental health during the study [43,46,47]. Two studies reported health impacts on children [41,45], whilst the other studies focused on adults: one study included older adults (aged 73 to 90 years) [43], and one study only included women [44]. Only one study was qualitative [43], whilst the remaining seven studies were quantitative.

There were six studies that reported on the effect of floods on participants with pre-existing depression. The risks of mental health problems following floods were observed over several different timeframes: ten days [41,45], two months [48], two to three months [42], and nine months [43]. Five studies showed that participants with pre-existing depression were at a significantly increased risk of depression [41,42,48], anxiety [48], PTSD [45], aggregated (depression, anxiety, and PTSD) [48], and of developing new symptoms [43] after a flood. For example, participants with pre-existing depression were at an 8.55-times-higher risk of depression following a flood [42]. A qualitative study about older adults within mental health services reported that five participants with pre-existing depression experienced either a deterioration in their mental health symptoms or the development of new symptoms, such as flashbacks about being evacuated up to nine months after the flood [43]. In contrast, two studies found no difference in symptoms following floods [44,48]. One study found no difference in depression or PTSD between women with and without pre-existing depression five months after a flood [44]. The second study also found no difference in PTSD after a flood between participants with and without pre-existing depression two months post-flood [48].

Only one study, which reported on women, investigated the effects of floods on participants with pre-existing anxiety [44]. The study found that women with pre-existing anxiety were at significantly increased risk of depression, anxiety, and PTSD after a flood [44]. Specifically, women with pre-existing anxiety were at a 9.85-times-higher risk of depression, 7.07-times-higher risk of anxiety, and 2.49-times-higher risk of PTSD compared with women without pre-existing anxiety five months after a flood [44].

In the qualitative study describing older adults within mental health services reported above [43], a deterioration in symptoms was found for one patient with chronic mixed anxiety and depression, triggered depression and preoccupations with the flood and perceived risk in one patient with pre-existing paranoid personality disorder, and a range of new mental health problems were reported for three patients with pre-existing dementia up to nine months after the flood.

Two studies reported on participants with pre-existing aggregated mental health problems (PTSD, major depression, panic disorder, generalised anxiety disorder, alcohol and drug misuse disorders [46]; PTSD, major depression, panic disorder, generalised anxiety disorder, alcohol and drug misuse disorders, and somatisation disorder [47]) and the health impacts after a flood. The studies found a significant difference between participants with pre-existing aggregated mental health problems and PTSD one to six months [46] and four months [47] after the flood. PTSD was found in three times as many participants with pre-existing aggregated mental health problems compared with those without pre-existing mental health problems (35% [46] and 34% [47] vs. 11% [46,47]). The studies also found that participants with pre-existing aggregated mental health problems were more likely to drink alcohol to cope with flooding (24% vs. 8%) [46] and develop new somatoform symptoms (somatic symptoms without a medical explanation) (37% vs. 16%) compared with participants without pre-existing mental health problems [47].

### 3.5. Wildfires

Seven studies reported on wildfires: six studies investigated impacts following the McMurray wildfire in Canada [50,51,52,53,54,55], and one study focused on a wildfire in Australia [49]. Three pre-existing mental health problems were investigated: depression [50,51,53,54,55], anxiety [50,51,53,54,55], and non-specified mental health problems [49,50,52] (Table 1). All seven studies reported on adults, with participants asked to retrospectively report on pre-existing mental health problems during the course of the study.

Five studies reported on the impact of wildfires on participants with pre-existing depression, with mixed findings. One study found that 18 months following a wildfire, participants with pre-existing depression were at a 4.63-times-higher risk of depression and 3.04-times-higher risk of anxiety compared with participants without depression [51]. The study also found a significant association between participants with pre-existing depression and PTSD after a wildfire, although there were no significant differences in risk compared with participants without pre-existing depression [51]. Similarly, two studies found no increased risk of depression for participants with pre-existing depression 6 months [55] and 18 months [54] after a wildfire. There was also no increased risk of anxiety for participants with pre-existing depression 6 months [50] and 18 months [54] after a wildfire, and no increased risk of PTSD for participants with pre-existing depression 6 months [53] and 18 months [54] after a wildfire.

Five studies investigated participants with pre-existing anxiety and exposure to wildfires, with mixed health impacts. First, one study found that participants with pre-existing anxiety were at a 5.13-times-higher risk of depression compared with participants without pre-existing anxiety six months after a wildfire [55]. In contrast, two studies reported no increased risk of depression for participants with pre-existing anxiety 18 months [51,54] after a wildfire, although one of the studies did report a significant association between participants with pre-existing anxiety and depression [51]. Second, two studies reported that participants with pre-existing anxiety were at a 6.76-times-higher risk of anxiety 6 months after a wildfire [50] and 2.66-times-higher risk 18 months after a wildfire [51] compared with participants without pre-existing anxiety. In contrast, one study found no difference in anxiety between participants with and without pre-existing anxiety 18 months after a wildfire [54]. Finally, one study found that participants with pre-existing anxiety were at a 7.89-times-higher risk of PTSD after 6 months [53] and another study found they were at a 5.80-times-higher risk of PTSD 18 months after a wildfire [51]. However, one study found no significant differences in PTSD between participants with PTSD and without PTSD 18 months after a wildfire [54].

There were three studies that investigated pre-existing non-specified mental health problems following exposure to wildfires. Evacuated participants with pre-existing non-specified mental health problems were at increased risk of depression, anxiety, PTSD, insomnia, and substance abuse 12 to 14 months after a wildfire compared with participants without non-specified mental health problems [52]. In addition, a significant increase in self-reported physical and mental health symptoms was found up to four months after an Australian wildfire [49]. However, two studies found that having pre-existing non-specified mental health problems did not lead to an increased risk of anxiety [50] or poor sleep [49] following exposure to a wildfire compared with those without mental health problems.

### 3.6. Wildfire and Flood/Cyclone

One study investigated participants who experienced a wildfire and flood/cyclone [56]. The study was in Australia and used the number of mental health therapy sessions attended following the wildfire and or flood/cyclone as an indicator of mental health impacts; pre-existing mental health was retrospectively measured during the study. A higher percentage of participants had sought mental healthcare after wildfires (42.7%) compared with those who had experienced floods/cyclones (30.2%). There were no significant differences between the total number of therapy sessions after a wildfire and flood/cyclone between participants who had accessed mental health services prior to the events and participants accessing services for the first time post-event (Table 1) [56].

### 3.7. Hurricanes

Two studies in the USA reported on the impact of hurricanes: one focused on participants with pre-existing PTSD [57] and the other on ADHD [58] (Table 1). Both studies retrospectively measured pre-existing mental health during the study.

The first study investigated a sub-sample of adult participants who had PTSD taken from a longitudinal study about people affected by a terrorist attack (11 September 2001) [57]. Participants who were subsequently affected by Hurricane Sandy in New York City were at a 6.6-times-higher risk of PTSD five to twelve months after the hurricane compared with participants unaffected by the hurricane.

The second study reported on children known to mental health services and affected by Hurricane Katrina in New Orleans [58]. Participants with ADHD were compared with participants with a range of mental health problems, which consisted of mood disorders, major depressive disorder, anxiety disorders, and conduct disorders. The study found that children with pre-existing ADHD were 365% more likely to be diagnosed with PTSD up to 28 months after a hurricane compared with children with aggregated mental health problems, excluding ADHD.

### 3.8. Drought

One study reported on drought [59]. The study focused on households in the USA (California) 22 months after the drought was declared a state of emergency. Participants were asked to self-report during an interview whether any members of their household had a mental health problem before the drought. A range of conditions were measured, such as whether the household had a private well and the number and age of people in the household. The study found no significant differences in the conditions measured and symptom deterioration in participants with and without non-specified pre-existing mental health problems (Table 1).

## 4. Discussion

This scoping literature review aimed to investigate the health impacts of climate-driven events on people with pre-existing mental health problems. While previous reviews identified associations between climate-driven events and impacts on mental health outcomes [5,6,7,8], to the best of our knowledge, this scoping review is the first to focus on people with pre-existing mental health problems. The review identified 31 studies that reported the health impacts of heat events, floods, wildfires, wildfire and flood/cyclone, hurricanes, and drought. In total, 90% of the included studies suggested that people with pre-existing mental health problems were particularly vulnerable to mental and physical health impacts following exposure to a climate-driven event. Where studies reported significant differences in health between people with and without pre-existing mental health problems, the impact on health for people with pre-existing mental health problems was always negative (e.g., increased mortality risk, increased risk of new symptom presentation, and exacerbation of symptoms): no positive impacts on health were reported. These findings highlight that people with pre-existing mental health problems may be considered as a particularly vulnerable population to the health impacts of climate-driven events.

For heat exposure events, four pre-existing mental health problems reported significant health impacts: schizophrenia (increased heatstroke risk) [37,40]; schizophrenia and comorbidities (increased heatstroke risk) [38,39]; dementia (increased mortality risk) [33,35]; and OCD (exacerbated symptoms) [36]. It would be pertinent to explore populations with other relevant pre-existing mental health problems, given the current evidence. Currently, the evidence is based on a small number of studies, warranting further research to solidify and expand our understanding. In terms of sample sizes and interpretation of findings, whilst the sample sizes for dementia and OCD studies were large (>3000 [35], >22,500 [33] and >700 [36], the schizophrenia studies [37,38,39,40] were case studies focusing on a single patient in each study, so the results of these case studies need to be interpreted with caution. There were three conditions where no significant impacts were reported: neurotic disorders; personality and behavioural disorders; and mania and bipolar, although these findings were from one study only [35]. For all other conditions, both significant and non-significant impacts were reported. However, as only two studies did not report any significant impacts, our findings suggest that people with pre-existing mental health conditions are vulnerable to the negative health impacts of heat events.

The review presents evidence that may demonstrate a difference in risk between people with and without pre-existing mental health problems. During heat events, vulnerable populations, such as the elderly and people with chronic physical health conditions, are at a heightened risk of experiencing negative health impacts, such as increased risk of mortality and heatstroke [5,8]. The review findings indicate that, following exposure to high temperatures, people with pre-existing mental health problems face similar negative health impacts, inclusive of increased risk of mortality [29,30,32,33,34,35], heatstroke [31,37,38,39,40] and exacerbation of existing health symptoms [36]. In addition, hospital admissions attributed to mental health issues are known to increase during heatwaves [60,61,62,63]. However, it is often not specified how many of these admissions are attributed to people with pre-existing mental health problems. The differences in health outcomes may be explained by several factors. Previous research suggests that psychiatric medications may also increase health risks, such as heightened heat risk associated with the use of hypnotic/anxiolytic and antipsychotic medications [33]; increased heat stroke and mortality risk associated with psychiatric medicine (amitriptyline, clozapine, or olanzapine) [30]; and inhibition of thermoregulation associated with antipsychotics, antidepressants, and mood-stabilising medicines [5]. Additionally, people with pre-existing mental health problems are particularly vulnerable to heat due to behavioural challenges in taking precautions to regulate their body temperature, such as drinking additional fluids, staying in cool areas, and wearing loose, light clothing [64,65].

This review found that the existing literature on flood events encompassed six pre-existing mental health problems, and significant impacts were reported for five of these pre-existing mental health problems: anxiety, chronic anxiety and mixed depression, paranoid personality disorder, dementia, and aggregated mental health problems. Previous research has found that people are at higher risk of PTSD, depression, and anxiety following a flood [66,67,68]. An important factor in reducing the adverse effects of flooding is accurate risk perception for people to be able to adapt their behaviours and move away from high-flood-risk areas [69]. In addition, low economic and personal resources can also prevent people from taking proactive flood-protection behaviours [69]. A combination of these factors is likely to impact people with mental health problems, as they commonly have fewer financial resources [19] and can be geographically restricted due to reliance on localised resources, such as access to medical treatment [20,21]. As a result, this population may have longer-term economic costs, which can lead to more adverse mental health issues. There were notable differences observed in the negative health impacts between different pre-existing mental health problems according to the amount of time expended after exposure to the flood event. Pre-existing anxiety was associated with an increased risk of depression, anxiety, and PTSD [44]. People with pre-existing chronic anxiety and mixed depression [43], paranoid personality disorder [43], and dementia [43] reported the presentation of new symptoms. Aggregated mental health problems were associated with increased risk of PTSD [46,47], substance misuse [46], and new symptom presentation [47]. People with pre-existing depression presented significant and non-significant health impacts; there were significantly increased risks of anxiety [48], aggregated mental health problems [48], and new symptom appearance [43], whilst significant and non-significant impacts on the risk of depression [41,42,44,48] and PTSD [44,45,48] were also reported. The findings currently indicate that people with pre-existing mental health problems are particularly vulnerable to the negative health impacts of floods; however, the small numbers of studies indicate that this area needs further investigation. 

A factor that may impact the risk of mental health problems after a flood is gender. Several studies have reported a greater risk of adverse health outcomes in females than males [68,70], with differences between how males and females reportedly respond to floods, with women experiencing a higher disruption to their sense of home and lower levels of self-confidence in being prepared for a flood than men [71,72]. One study in the review included an exclusively female sample, where participants with anxiety were at increased risk of depression, anxiety, and PTSD after a flood compared with women without pre-existing anxiety, although the difference was not significant [44]. However, the authors reported a possible error in how depression was measured, as the number of participants with depression decreased unexpectedly from 15% to 5% post-flood, which may explain these results. Thus, women with pre-existing anxiety may be more vulnerable to adverse mental health impacts after a flood than women with depression. However, these findings need to be interpreted with caution based on the methodology issues reported by the authors. Therefore, the literature suggests a potential emerging research area regarding the potential gender differences of people with pre-existing mental health problems and the ensuing health impacts of floods.

Wildfire events encompassed three pre-existing mental health problems: depression, anxiety, and non-specified problems. Non-specified mental health problems were associated with increased risk of depression, PTSD, and substance abuse [52], and exacerbation of physical and mental health symptoms [49]. Mixed findings were reported for people with pre-existing depression and anxiety, with both significant and non-significant impacts on health outcomes identified. One potential explanation for this trend may be the differences in the time points of the observation of impacts after a wildfire. Heightened health risks reportedly peaked 6 months after the event and decreased with time, but persisted up to 18 months and beyond [51]. This suggests that people experience the highest risk in the short-term period after an event; whilst the risk continues to diminish, it may not completely disappear, even up to 18 months later. Future research could benefit from exploring the influence of time expended after an event on people with pre-existing mental health problems, particularly pre-existing depression and anxiety.

The evidence identified for hurricanes consists of only two studies that investigated the effect of hurricanes on people with pre-existing mental health problems that largely focused on PTSD outcomes. There was an increased risk of PTSD for people with pre-existing PTSD twelve months after a hurricane [57]. However, it is difficult to draw conclusions about the longevity of these effects due to a lack of comparative studies with the same observation points and more long-term observation points. The second study found an increased risk of PTSD up to 28 months post-hurricane for people with ADHD, presenting long-term impacts for this group [53]. The longer-term impacts may potentially be higher, as suggested by a longitudinal study reporting delayed onset of PTSD to four years after the event [73], which may be moderated by stressful life events and a perceived lack of social support. The existing research begins to suggest that the effects of hurricanes on PTSD symptoms for some pre-existing mental health problems have complex prolonged, long-term effects. Future longitudinal research would benefit from examining the factors that affect the onset and severity of PTSD symptoms.

In terms of drought events, only one study included in the current review investigated pre-existing mental health and droughts, which found no significant impact on people with non-specified mental health problems [59]. Compared with heatwave and flood events, the health effects of droughts are relatively understudied; this could be due to challenges in determining drought start and end points. As a result, identifying and quantifying the health impacts of droughts are challenging, and may depend on many factors, including drought severity, population vulnerability, existing health and sanitation infrastructure, and available resources to mitigate impacts [74]. However, it is important to acknowledge that droughts could negatively impact those with pre-existing mental health problems, and some insight can be gained by examining the literature on the impacts of drought on mental health. Droughts negatively affect mental health in several ways, including stress/anxiety; trauma of witnessing damage/destruction of livestock and crops; decreased community resources/services/support systems; and social isolation as a result of loss of social networks/migration [75]. Studies based on Australian populations reported negative impacts of droughts on the mental health of farmers and farm workers; Aboriginal communities; rural communities; women; men; and adolescents [76,77,78,79,80,81,82,83]. It has been suggested that people exposed to droughts initially experience psychological distress, and after a threshold level of exposure of around 2.5–3 years, their distress levels begin to decrease [81]. As the study included in this review took place during the fourth year of a drought [59], it is possible that the threshold level had been reached and the distress of participants was reduced compared with if the study had been conducted during the first two years of the drought. These findings highlight that further work is required to understand how early exposure to drought (i.e., before the threshold described above) may affect those with mental health problems.

## 5. Limitations

Several limitations should be considered when interpreting the conclusions of this review. For clarity of reporting, we did not distinguish between mental health problems that were clinically diagnosed, were measured using a validated measurement tool, or recorded via self-reported history, retrospectively or before the event. When comparing the results, population information was not considered; certain characteristics, such as age, gender, income, and exposure to the event, may have impacted the results. In addition, whilst some studies included large sample sizes, they commonly only included relatively small numbers of people with mental health problems. Therefore, the conclusions from some studies should be interpreted with caution. The differences in severity between each climate-driven event were not reported, such as the length of the event, mortality, injury rates, and economic cost, all of which are likely to impact mental health.

It is important to note that several studies did not meet the inclusion criteria stating that the population included people with pre-existing mental health problems. However, these studies may have included people with pre-existing mental health problems, such as studies investigating hospital admissions for mental health issues and the connection between mental health medications and hospital admissions. Further studies are needed to collate adequate information in these settings so that the full depths of the impacts of climate-driven events on people with mental health problems can be identified.

## 6. Implications for Research and Practice

One of the difficulties in determining the impact of climate change on those with pre-existing mental health issues is the difficulty with measuring baseline values for mental health problems before a climate-driven event has occurred. As mentioned in the limitations, the quantification of the condition of pre-existing mental health problems relies on self-reported or clinical measures, which could range across various time points before the climate-driven event. Therefore, it is difficult to standardise time points amongst participants involved in relevant studies to create a baseline for pre-existing mental health problems, even within a single study. Furthermore, there would be considerable practical and ethical difficulties with sampling and measuring pre-existing mental health problems in a target population and exposing them to a climate-driven event. Considering the emerging importance of this vulnerable population that this review has highlighted, future research could benefit from using standardised measures of quantifying different pre-existing mental health problems to improve the comparability of findings across relevant studies.

With climate-driven events expected to increase in frequency and intensity in the future [9], further research is needed to explore different pre-existing mental health problems and the impacts of climate-driven events to create a framework that provides clear definitions of climate-driven events, the standardisation of measures for pre-existing mental health problems, the likely trajectory (e.g., health impacts at various time points), and the risk factors (e.g., characteristics of the individual and event) that impact the health effects. This will allow researchers to assess the impacts on health after a climate-driven event for people with and without pre-existing mental health problems.

The inclusion of more qualitative research would be a benefit to provide nuanced insight into the experience of health impacts relating to different pre-existing mental health problems and different climate-driven events. The majority of health outcomes were measured quantitatively by diagnostic tools, such as measuring the changes in anxiety symptoms before and after the event—qualitative methods could explore the broader impacts on health that quantitative measures or tools may not capture. Future research needs to include population factors, particularly investigating children, gender differences, and event exposure. In addition, future research should investigate advisory messaging aimed at people with mental health problems as an intervention to mitigate the worsening of their health problems.

Being prepared for climate-driven events is key to mitigating the mental health impacts following an event. Policies that include mental health need to be implemented at local and national levels to address climate change, covering specific information for people with pre-existing mental health problems and those who are at increased risk of poor mental health outcomes after a climate-driven event. For example, the World Health Organisation suggests that, to address the mental health impacts of climate change, governments should integrate climate considerations with mental health programmes; integrate mental health support with climate action; build upon global commitments; develop community-based approaches to reduce vulnerabilities; and close the large funding gap that exists for mental health and psychosocial support [84]. The public can also prepare for climate-driven events, which may reduce their worries or distress about climate change and mitigate adverse mental health outcomes after a climate-driven event; this can be through activism [85,86], creating a ‘go-bag’ (household items that may be needed in an emergency, such as water, cash, medicine, and personal identification) [87], and education about the risks of climate change specific to their health needs. In other words, an individual needs to be supported to be able to identify what is important to them in order to maintain their well-being and daily life, and to facilitate a plan to manage what they need to do if those factors are disrupted. Ideally, this should be a collaborative process with the individual and service providers (e.g., government and health professionals) so that the responsibility is not solely on the individual to manage; rather, systems can be adapted to suit and support them and the public in an emergency.

## 7. Conclusions

Research into the effect of climate-driven events on people with pre-existing mental health problems is limited. Most studies in the review reported on the health impacts among people with pre-existing mental health problems after a heat event, followed by flooding and wildfire. Further research is crucially needed to explore the full extent of the mental health risks of climate change among people with pre-existing mental health problems so that policymakers have information to be able to implement practices that can mitigate the risks and prevent the health gap from widening.

## Figures and Tables

**Table 1:** Summary of the health impacts after a climate-driven event among people with pre-existing mental health problems. Results from case studies are indicated with an asterisk (*). References are given in parentheses.

Climate-Driven Event	Pre-Existing Mental Health Problem	Impact on Health After a Climate-Driven Event (Health Problem)	No Significant Impacts on Health After a Climate-Driven Event	Inconclusive Impacts on Health After a Climate-Event
Heat events	Depression	Increased mortality risk [32,34,35]		Mortality risk [29]
	Psychosis	Increased mortality risk [34]	Mortality risk [33]	Mortality risk [29]
	Schizophrenia	Increased heatstroke risk [37,40] *		
	Schizophrenia and comorbidities	Increased heatstroke risk [38,39] *		
	Dementia	Increased mortality risk [33,35]		
	Substance misuse	Increased mortality risk [33]	Mortality risk [35]	
	Obsessive–compulsive disorder	Exacerbated symptoms (OCD) [36]		
	Neurotic disorders		Mortality risk [35]	
	Personality and behaviour disorders		Mortality risk [35]	
	Mania and bipolar		Mortality risk [35]	
	Non-specified ‡	Increased mortality risk [32]	Mortality risk [35]	
	Aggregated ‡	Increased mortality risk [30,33]	Mortality risk [35]	
		Increased heatstroke risk [31]		
Floods	Depression	Increased risk (depression) [41,42,48]	Depression [44]	
		Increased risk (anxiety) [48]		
		Increased risk (PTSD) [45]	PTSD [44,48]	
		New symptom presentation (non-specified) [43]		
		Increased risk (aggregated) [48]		
	Anxiety	Increased risk (depression) [44]		
		Increased risk (anxiety) [44]		
		Increased risk (PTSD) [44]		
	Chronic mixed anxiety and depression	New symptom presentation (non-specified) [43]		
	Paranoid personality disorder	New symptom presentation (non-specified) [43]		
	Dementia	New symptom presentation (non-specified) [43]		
	Aggregated ‡	Increased risk (PTSD) [46,47]		
		Increased risk (substance misuse) [46]		
		New symptom presentation (somatoform) [47]		
Wildfires	Depression	Increased risk (depression) [51]	Depression [54,55]	
		Increased risk (anxiety) [51]	Anxiety [50,54]	
			PTSD [53,54]	PTSD [51]
	Anxiety	Increased risk (depression) [55]	Depression [54]	Depression [51]
		Increased risk (anxiety) [50,51]	Anxiety [54]	
		Increased risk (PTSD) [51,53]	PTSD [54]	
	Non-specified ‡	Increased risk (depression) [52]		
		Increased risk (anxiety) [52]	Anxiety [50]	
		Increased risk (PTSD) [52]		
		Increased risk (insomnia) [52]	Poor sleep [49]	
		Increased risk (substance misuse) [52]		
		Exacerbated symptoms (non-specified, physical health) [49]		
		Exacerbated symptoms (non-specified, mental health) [49]		
Wildfire and flooding/cyclone	Non-specified ‡		Use of mental health services [56]	
Hurricanes	Post-traumatic stress disorder	Increased risk (PTSD) [57]		
	Attention-deficit hyperactivity disorder	Increased risk (PTSD) [58]		
Drought	Non-specified ‡		Non-specified [59]	

‡ ‘Non-specified’ and ‘aggregated’ mental health problems are defined in the study characteristics section of the methodology. Abbreviations: Obsessive–compulsive disorder (OCD); post-traumatic stress disorder (PTSD); attention-deficit hyperactivity disorder (ADHD). * highlights articles that are case studies

## Data Availability

All data relating to this study have been shared.

## Supplementary Materials

The following supporting information can be downloaded at: https://www.mdpi.com/article/10.3390/ijerph20085563/s1, Text S1: Search strategy.

## Appendix A

**Table A1:** Data extraction table for studies (n = 31) included in the scoping review.

Study	Country	Study Design	Total Participants n; Pre-Existing Mental Health (MH) n; Age (y = Years); % Male, % Female	Measurement/Definition of Pre-Existing Mental Health Problems Pre-Event	Measurement of Symptoms During and/or Post Event	Pre-Existing Mental Health (MH) Problem (‡)	Health Outcome	Effects on Health After the Climate-Driven Event
**Heat**								
De’donato, Stafoggia, Rognoni, Poncino, Caranci, Bisanti, Demaria, Forastiere, Michelozzi, Pelosini, and Perucci [29]	Italy	Quantitative case–crossover study	Adults Total n = 56,681; MH n = 588 (depression); 2038 (psychosis); Age = >35 y; Males = 48%; Females = 52%	Hospitalisation during the preceding two years (excluding the last 28 days)	Rome = four-year study period; Milan = five-year study period; Turin = seven-year study period	(i) Depression	(i) Mortality risk	(i) (a) Milan Airport: no increased risk (72% [95% CI −8–221]) (i) (b) Milan City: no increased risk (65% [95% CI −6–189]) (i) (c) Rome Airport: no increased risk (146% [95% CI 125–382]) **(i) (d) Rome City: increased risk (166% (95% CI 35–424)** (i) (e) Turin Airport: no increased risk (52% [95% CI −16–173]) (i) (f) Turin City: no increased risk (32% [95% CI −25–135])
						(ii) Psychosis	(ii) Mortality risk	**(ii) (a) Milan Airport: increased risk (157% [95%, CI 82–264])** **(ii) (b) Milan City: increased risk (91% [95% CI 40–161])** (ii) (c) Rome Airport: no increased risk (69% [95% CI 18–143]) (ii) (d) Rome City: no increased risk (82% [95% CI 28–158]) **(ii) (e) Turin Airport: increased risk (93% [95% CI 47–152]) ** (ii) (f) Turin City: no increased risk (73% [95% CI 32–126])
Kaiser, Rubin, Henderson, Wolfe, Kieszak, Parrott, and Adcock [30]	USA	Quantitative case–control study	Adults Total n = 51; MH n = 8; Age = 34–104 y; Males = 59%; Females = 41%	Reported retrospectively at the time of study	Twelve days during a heatwave	Aggregated (SZ, mentally handicapped, dementia, and alcohol abuse)	Mortality risk	**Increased mortality risk: OR = 14.0 (95% CI 1.8–633)**
Kim, Jo, Myung, and Jang [31]	South Korea	Quantitative case–control study	Children and adults Total n = 968; MH n = 261; Age = 0–19 y & 20–≥65 y; Males = 70%; Females = 30%	Reported retrospectively at the time of study	Four-month study period	Aggregated (SZ, dementia, depression, Parkinson’s disease, panic disorders, bipolar disorder, substance misuse, mental retardation, and unknown mental health diagnosis)	Heatstroke	(a) Increased heatstroke risk compared with mild heat illness: Fisher’s exact test, *p* < 0.001 (b) Increased heatstroke risk: adjusted OR = 7.69 (95% CI 4.06–14.54)
Naughton, Henderson, Mirabelli, Kaiser, Wilhelm, Kieszak, Rubin, and McGeehin [32]	USA	Quantitative case–control study	Adults Total n = 140; MH n = 36; Age = 35–93 y; Males = 49%; Females = 51%	Reported retrospectively at the time of study	Nine-day study period	(i) Depression	(i) Mortality risk	**(i) Increased mortality risk: OR = 4.1 (95% CI 1.3–12.5)**
						(ii) Non-specified (excluding depression)	(ii) Mortality risk	**(ii) Increased mortality risk: OR = 11.7 (95% CI 1.5–92.2)**
						(iii) Non-specified (including depression)	(iii) Mortality risk	**(iii) Increased mortality risk: OR = 5.7 (95% CI 1.9–16.8)**
Page, Hajat, Kovats, and Howard [33]	England	Quantitative cohort study	Unspecified Total n = 22,562; MH n = 22,562; Age = <65 y & ≥65 y; Males = unknown; Females = unknown	Registered diagnosis on medical records	Ten-year study period	(i) Psychosis	(i) Mortality risk	(i) No increased risk: RR = 1.02 (95% CI 0.95–1.09)
						(ii) Dementia	(ii) Mortality risk	**(ii) Increased risk of mortality for every 1 °C above 18 °C: RR = 1.03 (95% CI 1.00–1.07)**
						(iii) Alcohol misuse	(iii) Mortality risk	**(iii) Increased risk of mortality for every 1 °C above 18 °C: RR = 1.08 (95% CI 1.04–1.13)**
						(iv) Other substance misuse	(iv) Mortality risk	**(iv) Increased risk of mortality for every 1 °C above 18 °C: RR = 1.20 (95% CI 1.08–1.35)**
						(v) Aggregated (psychosis, dementia, and substance misuse)	(v) Mortality risk	**(v) Increased risk for mortality for every 1 °C above 18 °C: RR = 1.05 (95% CI 1.02–1.08)**
Stafoggia, Forastiere, Agostini, Biggeri, Bisanti, Cadum, Caranci, De’Donato, De Lisio, De Maria, Michelozzi, Miglio, Pandolfi, Picciotto, Rognoni, Russo, Scarnato, and Perucci [34]	Italy	Quantitative case–crossover study	Adults Total n =205,019; MH n = unknown; Age = ≥35 y; Males = 49%; Females = 51%	Hospital admission in the two years before death (excluding the last 28 days)	Three-to-six-year study period	(i) Depression	(i) Mortality	**(i) Increased risk of mortality at 30 °C compared with that at 20 °C: OR = 1.71 (95% CI 1.23–1.28)**
						(ii) Psychosis	(ii) Mortality	**(ii) Increased risk of mortality at 30 °C compared with that at 20 °C: OR = 1.70 (95% CI 1.39–2.09)**
Stivanello, Chierzi, Marzaroli, Zanella, Miglio, Biavati, Perlangeli, Berardi, Fioritti, and Pandolfi [35]	Italy	Quantitative case–crossover study	Adults Total n = 48,286; MH n = 3008; Age = 18–≥84 y; Males = 47%; Females = 53%	Accessed mental health service	Fourteen-year study period	(i) Mania and bipolar affective disorders	(i) Mortality risk	(i) No increased risk: OR = 1.03 (95% CI 0.88–1.20)
						(ii) Depression	(ii) Mortality risk	**(ii) Increased risk of mortality for every 1 °C above 24 °C: OR = 1.08 (95% CI 1.03–1.14)**
						(iii) Neurotic disorders	(iii) Mortality risk	(iii) No increased risk: OR = 0.99 (95% CI 0.90–1.08)
						(iv) Personal and behaviour disorders	(iv) Mortality risk	(iv) No increased risk: OR = 0.96 (95% CI 0.84–1.10)
						(v) Alcoholism and substance misuse	(v) Mortality risk	(v) No increased risk: OR = 0.96 (95% CI 0.72–1.29)
						(vi) Dementia and cognitive decline	(vi) Mortality risk	**(vi) Increased risk of mortality for every 1 °C above 24 °C: OR = 1.07 (95% CI 1.02–1.13)**
						(vii) Non-specified	(vii) Mortality risk	(vii) No increased risk: OR 0.90 (95% CI 0.67–1.21)
						(viii) Aggregated (SZ and other functional psychosis)	(viii) Mortality risk	(viii) No increased risk: OR = 1.05 (95% CI 0.95–1.16)
Brierley, Albertella, do Rosario, Ferrao, Miguel, and Fontenelle [36]	Brazil	Quantitative cross-sectional study	Adults Total n =742; MH n =742; Mean age =32.6 y; Males = 56%; Females = 54%	Reported retrospectively at the time of study	Six-year study period	OCD	OCD symptoms	**Exacerbation of OCD symptoms β = 0.153 [95% CI 1.49–4.30], (*p* < 0.001)**
Hoffmann, Oliveira, Lobato, and Belmonte-De-Abreu [37] *	Brazil	Qualitative case study	Adults Total n = 1; MH n = 1; Age = 60 y; Male = 100%	Onset of mental health problem aged 19 years	During event	SZ	Heatstroke	Glasgow Coma Scale 9/15, diagnosed with heat stroke, body temperature 41.9 °C
Kao and Kelly [38] *	Canada	Qualitative case study	Adults Total n = 1; MH n = 1; Age = 36 y; Male = 100%	History of mental health problems	During event	SZ and bipolar	Heatstroke	Glasgow Coma Scale 3/15, patient unresponsive and diagnosed with heat stroke, body temperature 42.2 °C
Kwok and Chan [39] *	Hong Kong	Qualitative case study	Adults Total n = 1; MH n = 1; Age = 48 y; Male = 100%	History of mental health problems	During event (two hospital admissions)	SZ and diabetes mellitus	Heatstroke	Admission 1: Glasgow Coma Scale 3/15, patient comatose, body temperature 42.4 °C Admission 2: Heat exhaustion (he was alert), body temperature 38.6 °C
Lee, Chen, and Chang [40] *	Taiwan	Qualitative case study	Adults Total n = 1; MH n = 1; Age = 49 y; Male = 100%	Onset of mental health problem aged 29 years	During event	SZ	Heatstroke	Glasgow Coma Scale 14/15, patient drowsy, weak, and slurring, body temperature 40.9 °C
**Flood**								
Felton, Cole, and Martin [41]	USA	Quantitative longitudinal study	Children Total n = 227; MH n = unknown; Age = 10–15 y; Males = unknown Females = unknown	Six months before	Ten days after	Depression	Depression	**Association: *r* = 0.72, *p* < 0.001**
Ginexi, Weihs, Simmens, and Hoyt [42]	USA	Quantitative longitudinal study	Adults Total n = 1735; MH n = unknown Age = 18–98 y; Males = 34%; Females = 66%	One year before	Sixty to ninety days after	Depression	Depression	**(a) Association: *r* = 0.54, *b* = 0.51, *p* < 0.001** **(b) Increased depression risk: OR = 8.55 (95% CI 5.54–13.21); *r* = 0.35, *b* = 2.15, *p* < 0.001 **
Hayes, Mason, Brown, and Mather [43]	England	Qualitative study	Elderly Adults Total n = 87; MH n = 87; Age = 73–90 y; Males = 41%; Females = 59%	Accessed mental health services	Immediate and up to nine months after	(i) Dementia	(i) Changes in symptoms and/or behaviour	(i) (a) Appearance of new symptoms (i) (b) Exacerbation of symptoms
						(ii) Depression	(ii) Changes in symptoms and/or behaviour	(ii) (a) Appearance of new symptoms (ii) (b) Exacerbation of symptoms
						(iii) Paranoid personality disorder	(iii) Changes in symptoms and/or behaviour	(iii) Appearance of new symptoms
						(iv) Chronic mixed anxiety and depression	(iv) Changes in symptoms and/or behaviour	(iv) Exacerbation of symptoms
Hetherington, McDonald, Wu, and Tough [44]	Canada	Quantitative longitudinal study	Women Total n = 923; MH n = unknown; Age (mean) = 34.5 y; Women = 100%	Differed between participants, up to 18–36 months before	Five months after	(i) Depression	(i) (a) Depression	(i) (a) No increased risk: adjusted OR = 1.15 (95% CI 0.47–2.82)
							(i) (b) PTSD	(i) (b) No increased risk: adjusted OR = 1.37 (95% CI 0.62–3.07)
						(ii) Anxiety	(ii) (a) Depression	**(ii) (a) Increased risk: adjusted OR = 9.85 (95% CI 4.06–23.96)**
							(ii) (b) Anxiety	**(ii) (b) Increased risk: adjusted OR = 7.07 (95% CI 4.36–11.45)**
							(ii) (c) PTSD	**(ii) (c) Increased risk: adjusted OR = 2.49 (95% CI 1.17–5.26)**
Martin, Felton, and Cole [45]	USA	Quantitative longitudinal study	Children Total n = 125; MH n = unknown; Age = 10–15 y; Males = unknown; Females = unknown	Six months before	Ten days after	Depression	PTSD	**(a) Association: *r* = 0.35, *p* < 0.001** **(b) Significantly predicted PTSD symptoms: *β* = 0.24, *p* < 0.05.**
McMillen, North, Mosley, and Smith [46]	USA	Quantitative cross-sectional study	Adults Total n = 162; MH n = 71; Age (mean) = 49.5 y; Males = 34%; Females = 66%	Reported retrospectively at the time of study (one month to at least four years prior)	One to six months after	Aggregated (PTSD, major depression, panic disorder, generalised anxiety disorder, and alcohol and drug misuse disorders)	(i) PTSD	**(i) Increased risk: 34% compared with 11% without a history of mental health problems (*x^2^ (1)* = 12.27, *p* = 0.001)**
							(ii) Substance misuse	**(ii) Increased alcohol misuse risk: 24% compared with 8% without a history of mental health problems (*x^2^* (1) = 7.96, *p* = 0.005)**
North, Kawasaki, Spitznagel, and Hong [47]	USA	Quantitative longitudinal study	Adults Total n = 162; MH n = 72; Age = 18–≥65 y; Males = 33%; Females = 67%	Reported retrospectively at the time of study	Four months and sixteen months post	Aggregated (PTSD, major depression, panic disorder, generalised anxiety disorder, alcohol and drug misuse disorders, and somatization disorder)	(i) PTSD	**(i) Increased risk: 35% compared with 11% without a history of mental health problems at four-month follow-up (*x^2^* (1) = 13.17, *p* < 0.001)**
							(ii) Somatoform symptoms	**(ii) Increased risk of developing new somatoform symptoms: 37% compared with 16% without a history of mental health problems (x^2^ (1) = 9.79, *p* < 0.002)**
Hoffman [48]	USA	Quantitative longitudinal study	Adults Total n = 463; MH n = 463; Age = 19–89 y; Males = 32%; Females = 68%	One year before	Two months after	(i) Depression	(i) Depression	**(i) Association: *β* = 0.41, *p* < 0.001**
							(ii) Anxiety	**(ii) Association: *β* = 0.38, *p* < 0.001**
							(iii) PTSD	(iii) No increased risk: *β* = 0.05, *p* > 0.05
							(iv) Aggregated (depression, anxiety, and PTSD)	**(iv) Association: *β* = 0.421, *p* < 0.001**
**Wildfires**								
Rodney, Swaminathan, Calear, Christensen, Lal, Lane, Leviston, Reynolds, Trevenar, Vardoulakis, and Walker [49]	Australia	Quantitative cross-sectional survey	Adults Total n = 2084; MH n = 441; Age = 18–85 y; Males =40%; Females = 60%	Reported retrospectively at the time of study	Two weeks to four months	Non-specified	(i) Mental health symptoms	**(i) Increased worsening symptoms risk: adjusted OR = 1.30 (1.01–1.66), *p* = 0.038**
							(ii) Physical health symptoms	**(ii) Increased physical symptoms risk: adjusted OR = 1.64 (95% CI 1.28–2.09), *p* < 0.001 **
							(iii) Poor sleep	(iii) No increased risk: OR = 1.19 (95% CI 0.95 to 1.50), *p* = 0.133
Agyapong, Hrabok, Juhas, Omeje, Denga, Nwaka, Akinjise, Corbett, Moosavi, Brown, Chue, Greenshaw, and Li [50]	Canada	Quantitative cross-sectional survey	Adults (n = 486, 18–≥40 y, 33%, 67%) 103 with mental health problems	Reported retrospectively at the time of study	Six months after	(i) Depression	(i) Anxiety	(i) No increased risk: OR = 3.26 (95% CI 0.97–10.95)
						(ii) Anxiety	(ii) Anxiety	**(ii) Increased risk for GAD: OR = 6.76 (95% CI 1.65–27.70)**
						(iii) Non-specified	(iii) Anxiety	(iii) No increased risk: OR = 2.81 (95% CI 0.56–14.01)
Moosavi, Nwaka, Akinjise, Corbett, Chue, Greenshaw, Silverstone, Li, and Agyapong [51]	Canada	Quantitative cross-sectional survey	Adults Total n = 290; MH n = 66; Age = ≥18 y; 45%, 55%	Reported retrospectively at the time of study	18 months after	(i) Depression	(i) (a) Depression	**(i) (a) (a) Association: *x^2^* = 0.36, *p* < 0.001** **(i) (a) (b) Increased risk: OR = 4.63 (95% CI 1.77–12.12), *p* < 0.001**
							(i) (b) Anxiety	**(i) (b) (a) Association: *x^2^* = 0.34, *p* < 0.001 ** **(i) (b) (b) Increased risk: OR = 3.04 (95% CI 1.21–7.61), *p* = 0.02**
							(i) (c) PTSD	**(i) (c) (a) Association: *x^2^* = 0.31, *p* < 0.001** (i) (c) (b) No increased risk: OR = 1.73 (95% CI 0.56–5.39), *p* = 0.34
						(ii) Anxiety	(ii) (a) Depression	**(ii) (a) (a) Association: *x^2^* = 0.29, *p* < 0.001** (ii) (a) (b) No increased risk: OR = 1.28 (95% CI 0.47–3.53), *p* = 0.63
							(ii) (b) Anxiety	**(ii) (b) (a) Association: *x^2^* = 0.30, *p* < 0.001** **(ii) (b) (b) Increased risk: OR = 2.68 (95% CI 1.04–6.89), *p* = 0.04**
							(ii) (c) PTSD	**(ii) (c) (a) Association: *x^2^* = 0.34, *p* < 0.001** **(ii) (c) (b) Increased risk: OR = 5.80 (95% CI 1.92–17.50), *p* = 0.002**
Belleville, Ouellet, Lebel, Ghosh, Morin, Bouchard, Guay, Bergeron, Campbell, and MacMaster [52]	Canada	Quantitative cross-sectional survey	Adults Total n = 1510; MH n = 177; Age = ≥18 y; Males = 44%; Females = 56%	Reported retrospectively at the time of study	12 to 14 months after	Non-specified	(i) Depression	**(i) Increased risk: adjusted *β* = 2.98 (95% CI 2.31–3.65), *p* < 0.0001**
							(ii) Anxiety	**(ii) Increased risk: adjusted *β* = 2.15 (95% CI 1.51–2.80), *p* < 0.0001**
							(iii) PTSD	**(iii) Increased risk: adjusted *β* = 3.94 (95% CI 2.21–5.66), *p* < 0.0001**
							(iv) Insomnia	**(iv) Increased risk: adjusted *β* = 2.26 (95% CI 1.38–3.13), *p* < 0.001**
							(v) Substance use	**(v) Increased risk: adjusted *β =* 0.33 (0.18–0.48), *p* < 0.0001**
Agyapong, Juhas, Omege, Denga, Nwaka, Akinjise, Corbett, Brown, Chue, Li, and Greenshaw [53]	Canada	Quantitative cross-sectional survey	Adults Total n = 486; MH n = 103; Age = 16–88 y; Males = 34%; Females = 66%	Reported retrospectively at the time of study	Six months after	(i) Depression (ii) Anxiety	(i) PTSD (ii) PTSD	(i) No increased risk: OR = 1.78 (95% CI 0.53–6.03) **(ii) Increased risk: OR = 7.89 (95% CI 1.70–36.59)**
Agyapong, Ritchie, Brown, Noble, Mankowsi, Denga, Nwaka, Akinjise, Corbett, Moosavi, Chue, Li, Silverstone, and Greenshaw [54]	Canada	Quantitative cross-sectional survey	Adults Total n = 197; MH n = unknown; Age = ≥18 y; Males = 15%; Females = 85%	Reported retrospectively at the time of study	18 months after	(i) Depression	(i) (a) Depression	(i) (a) No increased risk: *x^2^* = 0.13 (*p* = 0.14)
							(i) (b) Anxiety	(i) (b) No increased risk: *x^2^* = −0.01 (*p* = 1.0)
							(i) (c) PTSD	(i) (c) No increased risk: *x^2^* = 0.02 (*p* = 1.0)
						(ii) Anxiety	(ii) (a) Depression	(ii) (a) No increased risk: *x^2^* = −0.005 (*p* = 1.0)
							(ii) (b) Anxiety	(ii) (b) No increased risk: *x^2^* = −0.12 (*p* = 0.15)
							(ii) (c) PTSD	(ii) (c) No increased risk: *x^2^* = 0.02 (*p* = 0.69)
Agyapong, Juhas, Brown, Omege, Denga, Nwaka, Akinjise, Corbett, Hrabok, Li, Greenshaw, and Chue [55]	Canada	Quantitative cross-sectional survey	Adults Total n = 486; MH n = 103; Age = 18–≥40 y; Males = 34%; Females = 66%	Reported retrospectively at the time of study	Six months after	(i) Depression	(i) Depression	(i) No increased risk: OR = 1.10 (95%, CI 0.34–3.55)
						(ii) Anxiety	(ii) Depression	**(ii) Increased risk: OR = 5.13 (95% CI 1.31–20.12)**
**Wildfire and flood/cyclone**								
Reifels, Bassilios, Spittal, King, Fletcher, and Pirkis [56]	Australia	Quantitative cross-sectional study	Unclear Total n = 2693; MH n = 1042; Mean = 41 y; Males = 39%; Females = 61%	Reported retrospectively at the time of study	Two-year study period	Non-specified	Number of mental health therapy sessions	(a) No increased risk: OR = 0.95 (95% CI 0.80–1.13), *p* = 0.583 (b) Number of sessions per mental health referral: 4.87 (95% CI 4.38 to 5.36) compared with 5.11 (95% CI 4.39 to 5.83) without a history of mental health problems
**Hurricanes**								
Caramanica, Brackbill, Stellman, and Farfel [57]	USA	Quantitative longitudinal survey	Adults Total n = 4137; MH n = 335 Age = ≥18 y; Males = 56%; Females = 43%	Seven to 17 months before	Five to 12 months after	PTSD	PTSD	**Increased PTSD risk: adjusted OR = 6.6 (95% CI 4.6–9.6)**
Airhia [58]	USA	Quantitative cross-analysis	Children Total n = 77; MH n = 77; Age = 5–19 y; Males = 65%; Females = 35%	Zero to 22 months before	Zero to 28 months after	ADHD	PTSD	**Increased PTSD risk: *β* = 1.54, *p* = 0.049**
**Drought**								
Barreau, Conway, Haught, Jackson, Kreutzer, Lockman, Minnick, Roisman, Rozell, Smorodinsky, Tafoya, and Wilken [59]	USA	Quantitative cross-sectional study	Households Total n = Unknown; MH n = 2742; Age = ≤18–≥65 y; Males = Unknown; Females = Unknown	Reported retrospectively at the time of study	22 months after drought state of emergency	Non-specified	Worsening of symptoms	No increased risk: (a) Household did not have running water: weighted OR = 0.83 (95% CI 0.13–5.54) (b) Household had private well before drought: weighted OR = 1.19 (95% CI 0.31–4.60) (c) Negatively affected household finances: weighted OR = 0.58 (95% CI 0.16–1.62) (d) Negatively affected household property: weighted OR = 1.02 (95% CI 0.31–3.34)

‡ When the study did not specify the mental health problem they investigated, we defined it as “non-specified.” When the study specified multiple mental health problems, although they were grouped into one variable, we defined the mental health problem as “aggregated;” the mental health problems included in the variable are reported in parentheses. * Case studies; Bold text represents significant results. Glasgow Coma Scale: Coma severity out of 15 points (Eye (4), Verbal (5), Motor (6)), 0 = High severity to 15 = Low severity. Abbreviations: Odds ratio (OR); relative risk (RR); obsessive compulsive disorder (OCD); post-traumatic stress disorder (PTSD); generalised anxiety disorder (GAD); major depressive disorder (MDD); schizophrenia (SZ); attention-deficit hyperactivity disorder (ADHD).
