# Supplementary material for: Investigating the Health Impacts of Climate Change among People with Pre-Existing Mental Health Problems: A Scoping Review
Source: Int J Environ Res Public Health. 2023 Apr 18;20(8):5563. doi: 10.3390/ijerph20085563 (PMC10138675; doi:10.3390/ijerph20085563)
Supplement: Supplementary file 1 [file ijerph-20-05563-s001.zip › ijerph-2222581-supplementary.pdf]

Embase, Medline, APA PsycInfo

1. Exp climate.mp
2. “global warming”.mp
3. “green house”.mp
4. “extreme weather”.mp
5. “hot weather”.mp
6. “heat wave*”.mp
7. “extreme heat”.mp
8. “forest fire*”.mp
9. “bush fire*”.mp
10. “wildfire*”.mp
11. flood*.mp
12. drought*.mp
13. “crop failure*”.mp
14. famine*.mp
15. “displaced population”.mp
16. 1 OR 2 OR 3 OR 5 OR 6 OR 7 OR 8 OR 9 OR 10 OR 11 OR 12 OR 13 OR 14 OR 15
17. pre-existing.mp
18. prior.mp
19. previous.mp
20. 17 OR 18 OR 19
21. “mental health”.mp
22. “psychological disorder”.mp
23. “psychological health”.mp
24. depression*.mp
25. anxiety.mp
26. “post traumatic stress”.mp
27. “personality disorder”.mp
28. psycho*.mp
29. schiz*.mp
30. well-being.mp
31. 20 OR 21 OR 22 OR 23 OR 24 OR 25 OR 26 OR 27 OR 28 OR 29 OR 30
32. 16 AND 31
